# Perspective on dysphagia screening, assessment methods, and protocols in intensive care units: an opinion article

**DOI:** 10.3389/fnhum.2024.1375408

**Published:** 2024-04-09

**Authors:** Maria Demetriou, Anastasios M. Georgiou

**Affiliations:** The “Cyprus Rehabilitating Aphasia and Dysphagia” (C-RAD) Lab, Department of Rehabilitation Sciences, School of Health Sciences, Cyprus University of Technology, Limassol, Cyprus

**Keywords:** intensive care unit (ICU), dysphagia, screening tools, assessment practices, protocol and guidelines

## 1 Introduction

Intensive care unit (ICU) admission and related prolonged intubation has been identified as a substantial risk factor for the development of swallowing problems (aka dysphagia) (Perren et al., 2019; Spronk et al., 2022; Royals et al., 2023). Indeed, post-extubation dysphagia (PED) has a documented prevalence rate of 93% (Macht et al., 2013) and has been linked to adverse health outcomes and risks, including aspiration-related pneumonia (Barker et al., 2022; Freeman-Sanderson et al., 2023; Royals et al., 2023), malnutrition (Barker et al., 2022; Royals et al., 2023), dehydration (Royals et al., 2023), re-intubation (Muñoz-Garach et al., 2023; Royals et al., 2023), prolonged mechanical ventilation (MV) and length of ICU/hospital stay (Barker et al., 2022; Muñoz-Garach et al., 2023; Royals et al., 2023; Clayton et al., 2024). Additionally, it contributes to delayed recovery (Royals et al., 2023), reduced quality of life (QoL), and higher short-term (28 days) and mid-term (90 days) mortality rates (Perren et al., 2019; Muñoz-Garach et al., 2023; Clayton et al., 2024).

Despite being prevalent and clinically significant in the ICU, PED remains under-recognized due to minimal routine screening (Zurbano et al., 2023) and a lack of comprehensive assessment guidelines (Likar et al., 2024). Fewer than thirty percent of surveyed ICU practitioners employ dysphagia protocols, while less than 20% of nurses undergo formal dysphagia training regarding the screening of patients for suspected PED (Freeman-Sanderson et al., 2023). But dysphagia screening and thereafter assessment is particularly crucial for its early identification, management, prevention and mitigation of complications, optimization of nutritional support and ultimately enhancement of patient overall health outcomes (Freeman-Sanderson et al., 2023; Mpouzika et al., 2023; Troll et al., 2023; Zurbano et al., 2023).

This opinion article explores dysphagia screenings, assessment methods and protocols used in the ICU setting for PED, highlighting the urgent need for innovative approaches, while also addressing long-term challenges and suggesting potential actions.

## 2 Current landscape of dysphagia screening and assessment in ICUs

The current landscape of dysphagia assessment in the ICU setting is characterized by a variety of screening and clinical examination methods and protocols. However, in the existing body of literature validated non-instrumental dysphagia screening and assessment methods specifically developed for ICU patients are conspicuously absent (Spronk et al., 2022). While evaluation to detect potential swallowing difficulties in acute stroke patients is strengthened and supported by guidelines and internationally standardized protocols (Dziewas et al., 2021), the development and use of equivalent protocols and guidelines for dysphagia screening and assessment appears to be marginalized in the ICU context (Macht et al., 2013). Water swallow tests and multi-consistency screenings are the two methods for screening ICU patients for suspected PED (Likar et al., 2024), while methods used to assess dysphagia can be classified under two broad categories: instrumental and non-instrumental. It is usually accepted that these two modalities should complement one another for a comprehensive understanding of dysphagia (Perren et al., 2019; Troll et al., 2023; Likar et al., 2024).

### 2.1 Dysphagia screening tools

With regards to non-instrumental PED testing, a recent systematic review conducted by Perren et al. (2019) showed considerable heterogeneity among relevant screening and assessment tools, and their components, and structure; highlighting the paucity of studies focusing on ICU dysphagia. So far, validated and reliable PED screening tools in heterogeneous non-specific cohorts incorporate a water swallowing test (Likar et al., 2024). Some of these include the Postextubation Dysphagia Screening (PEDS) tool (Johnson et al., 2018), the Yale Swallow Protocol (Suiter et al., 2014), the 3-ounce water swallow test (Suiter and Leder, 2007), the Volume-Viscosity Swallow Test (V-VST) (Clavé et al., 2008), and the Bernese ICU Dysphagia Algorithm (Zuercher et al., 2020). Although Leder and Suiter (2014) recommend the Yale Swallow Protocol as a bedside tool to routinely screen medical and surgical ICU patients in terms of aspiration risk, the validation of administering this tool to ICU patients has not yet been conducted. Similarly, despite the 3-ounce water swallow test not being validated in critically ill patients, its use is recommended to be performed by ICU nursing personnel, given that patients are alert and capable of sitting upright in a supported position (Macht et al., 2014). The Volume-Viscosity Swallow Test (V-VST) has demonstrated acceptable sensitivity and specificity in mixed and specific patient populations, but not specifically in critically ill patients (Perren et al., 2019; Troll et al., 2023) as limited evidence exists regarding its applicability in ICU patients (Zurbano et al., 2023). The Gugging Swallowing Screen (GUSS) is also included in the spectrum of clinical non-instrumental screenings aimed at specific patient populations (Perren et al., 2019; Troll et al., 2023). Despite Muñoz-Garach et al. (2023) arguing that GUSS may be suitable for dysphagia screening in the ICU, its validity has only been established for stroke patients (Trapl et al., 2007). Schefold et al. (2017) advocate the adoption of a Systematic Bedside Screening Algorithm, which includes the Bedside Swallowing Evaluation (BSE) and the Water Swallowing Test (WST) for critically ill patients. Nonetheless, both their validity and reliability are considered ambiguous, especially when it comes to their usage and application for the detection and evaluation of suspected PED in ICU patients (Spronk et al., 2022).

Currently, the only non-instrumental screening tools that have been formally piloted and validated in the ICU population so far are the Postextubation Dysphagia Screening (PEDS) tool by Johnson et al. (2018), and the Bernese ICU Dysphagia Algorithm the validation of which is underway (Likar et al., 2024). Furthermore, a recently proposed decision-making guideline entitled Swallowing Algorithm Post-Extubation (SAPE) (Barker et al., 2022), which was established to systematically, timely and thoroughly screen ICU patients for suspected PED, showed effective application and usage in several ICUs, thus indicating the usefulness and benefit of such a guideline for PED screening. Nonetheless, validation in a heterogeneous ICU population is still necessary. The Bedside Swallowing Evaluation Decision Tree Algorithm (Moss et al., 2020) is considered another algorithm, suggesting that it could potentially serve as a useful clinical screening test for detecting aspiration in survivors of acute respiratory failure (ARF). However, this tool also requires validation with ARF survivors.

### 2.2 Dysphagia assessment tools

Several studies support the use of Videofluoroscopic Swallow Study (VFSS) and Fiberoptic Endoscopic Evaluation of Swallowing (FEES) (Perren et al., 2019; Barker et al., 2022; Freeman-Sanderson et al., 2023; Mpouzika et al., 2023; Troll et al., 2023; Likar et al., 2024) as the designated reference-standard for the assessment of dysphagia, with FEES constituting the most preferred method within the ICU setting due to its accessibility (Likar et al., 2024). Nonetheless, instrumental examinations are not always widely available, readily accessible, or feasible (Barker et al., 2022).

Recently, Mpouzika et al. (2023) reported that the cough reflex test and the water swallow test are the most frequently methods used for the evaluation of PED in ICU environments. The precision of these commonly employed methods though, is highly questionable as to whether they assist in the accurate detection of PED as well as their contribution to prevent silent aspiration, which in turn is likely to predispose patients to pneumonia. The use of ice chips as a method to evaluate and rehabilitate impaired swallowing function is described as an alternative protocol outlined by Pisegna and Langmore (2018). However, to ascertain its safety, effectiveness, and related outcomes, this protocol needs to be systematically investigated. Other assessment methods and tools, including the clinical Bedside Swallowing Evaluation (BSE) performed by speech and language pathologists (SLPs), the Mann Assessment of Swallowing Ability (MASA) (Mann, 2002), and the McGill Ingestive Swallowing Assessment (MISA) (Lambert et al., 2003) are widely used for the evaluation of swallowing disorders in diverse hospitalized populations. With regards to ICU patients the BSE has shown variable accuracy in detecting aspiration (Lynch et al., 2017). The Mann Assessment of Swallowing Ability (MASA), which was initially validated in stroke patients and subsequently in mixed populations, is not advocated for use in mixed populations (Perren et al., 2019). Likewise, the application of the McGill Ingestive Swallowing Assessment in severely affected patients is not recommended (Perren et al., 2019). On the other hand, the Danish version of the McGill Ingestive Swallowing Assessment (MISA-DK) (Hansen et al., 2010) has been validated in a mixed population of acute hospitalized patients with high internal consistency (Perren et al., 2019).

Despite the wide range of methods used for both screening and evaluating PED in the ICU setting, not all of these methods have been validated for use in this particular setting. This raises doubts and concerns about their effectiveness, reliability, and validity which are all essential for the assurance of patient safety, holistic and individualized care, improvement of the quality of patient care, and overall health outcomes.

## 3 Validated tools for PED screening in the ICU

In the most recent literature, the only examples of multi-consistency screening that have been studied and validated in ICU populations are the Gugging Swallowing Screen for ICU (Troll et al., 2023) and the modified Volume Viscosity Test (Zurbano et al., 2023).

### 3.1 Gugging swallowing screen for ICU (GUSS-ICU)

The GUSS-ICU represents a simple, reliable, and valid multi-consistency bedside swallowing test developed to accurately identify PED within ICUs. It is intended as a supplemental tool, designed for use alongside instrumental swallowing diagnostics and clinical assessments performed by SLPs (Troll et al., 2023). The tool is a modification of the original GUSS (Trapl et al., 2007) and consists of two parts. In the first part, six items are assessed: the Richmond Agitation Sedation Scale score (from 0 to +2), the presence of stridor, the effectiveness of cough strength and throat clearing, the presence of drooling, the ability of saliva swallowing, and the voice alteration after saliva swallow. Any non-detected item receives one point. A prerequisite for the patient to proceed to the second half is success in obtaining all 6 points of the first half, otherwise the procedure is discontinued. The second part requires four consecutive swallowing trials of levels 3, 0, and 7 of the International Diet Standardization Initiative (IDDSI) (Cichero et al., 2017; International Dysphagia Diet Standardization Initiative and Framework Testing Methods, 2019) and finishes with a mixed solid-liquid consistency. During the swallowing trials, swallowing difficulties, coughing, drooling, or voice changes are observed. Each of these is scored with one point. If an abnormal swallow is observed in any of the 4 evaluation criteria, this criterion is given a score of 0 and the test is terminated. A patient's swallowing function may be scored as normal without risk of aspiration if they score the maximum score of 10 points. Depending on each patient's score on this screening test, appropriate dietary recommendations are given. The validity of the GUSS-ICU was assessed using FEES as a reference standard and demonstrated a sensitivity rate between 89% and 92% and a specificity rate of 67% to 89%, while inter-rater reliability was strong (Troll et al., 2023).

We recommend the use of GUSS-ICU as a screening tool for PED in the ICU setting. Beyond its ability to accurately detect PED in critically ill ICU patients, it has a multi-consistency nature that also allows for the provision of personalized dietary recommendations depending on the score achieved by each patient. This way, the risk of penetration and/or aspiration before, during, and after swallowing is reduced. Although the GUSS-ICU has not yet been validated for use by nursing staff, we suggest that it could be employed by ICU nurses for the purpose of the assurance of systematic dysphagia screening when dysphagia specialists are either unavailable or not part of ICU teams.

### 3.2 Modified volume-viscosity swallow test (mV-VST)

The research study conducted by Zurbano et al. (2023) confirms that the modified Volume-Viscosity Swallow Test (mV-VST) is an efficient bedside tool for detecting aspiration among extubated and tracheostomized critically ill patients. The tool is also a multi-consistency dysphagia screening tool that is based on swallowing trials of various volumes and viscosities. Testing starts with nectar viscosity, followed by pudding and then liquid viscosities (Zurbano et al., 2023). The patient receives sequentially increasing volumes of 5, 10 and 15 ml for each viscosity, with the use of a 50 ml syringe (Laguna et al., 2022; Zurbano et al., 2023). For each viscosity, there are four safety parameters evaluated: the presence of cough, desaturation >3%, altered voice tone, and the presence of pharyngeal debris (Laguna et al., 2022; Zurbano et al., 2023). The viscosities shall be assessed gradually, as long as no changes in the above parameters are detected. The evaluation is discontinued if the patient shows unsafe swallowing on any volume or viscosity trial. Conversely, when the patient demonstrates safe swallowing on all trials, suspicions of dysphagia and aspiration are ruled out (Zurbano et al., 2023). The mV-VST is capable of detecting aspiration with a specificity rate of 72% and a sensitivity rate of 89.5% in decannulated patients, with a negative predictive value of 90%, indicating a highly likelihood of identifying false-positive tests (Zurbano et al., 2023). Surprisingly, mV-VST detects aspiration with a perfect sensitivity score and a negative predictive value of 100% and a specificity rate of 78.8% in tracheostomized patients (Zurbano et al., 2023). Hence, the mV-VST tool is characterized as a valid method for detecting aspiration in intubated and tracheostomized patients, demonstrating superior performance in identifying dysphagia among tracheostomized patients.

We also encourage the use of this non-instrumental dysphagia screening tool in an effort to minimize the risk of aspiration-related complications in ICU patients and to provide appropriate dietary guidance, particularly for tracheostomized patients. Nonetheless, healthcare professionals in the ICU setting should be extremely careful when administering this test to extubated patients as false-positive results may occur. Additional instrumental evaluations would be necessary in this scenario.

## 4 Evolving dysphagia screening and assessment methods in the ICU

From the above analysis it is evident that there is an urgent need for the establishment of universally recognized dysphagia screening and assessment protocols in ICUs. Advancements in such methods will contribute to increased precision in evaluating PED traits and progression. Standardizing screening as well as assessment protocols across diverse healthcare settings both locally and internationally will (i) promote consistent application of diagnostic criteria and (ii) enhance the reliability comparisons and data interpretation. The adoption of such standardized protocols not only would promote effective communication and collaboration among healthcare professionals, but also, when combined with instrumental methods, would facilitate the development of innovative dysphagia screening and assessment tools for critically ill patients. This strategic approach is recommended to strengthen diagnostic accuracy, guide effective and personalized treatments and this way improve the overall standard of ICU PED related care while reducing hospital costs.

## 5 Discussion

The absence of internationally standardized protocols and evidence-based clinical guidelines for PED screening and assessment has been a long-standing barrier within ICUs (Armas-Navarro et al., 2023; Freeman-Sanderson et al., 2023). Recognizing the current gap, dysphagia professionals are urged to intensify their research efforts and focus on establishing guidelines that will facilitate the diagnosis, prevention, and management of PED in the complex context of ICUs. In support of the commitment to create such guidelines, it seems more practical to establish an internationally standardized, recognized, effective and patient-centered framework. This step is essential for several reasons. First, it will reduce the considerable burden of PED in critically ill patients, thereby ensuring the implementation of safe clinical practices. Secondly, it will ensure consistency in PED assessment across different regions around the globe, fostering consistency and comparability of outcomes. Thirdly, it will increase screening, assessment and treatment effectiveness by providing evidence-based approaches. Furthermore, as dysphagia is culture-free, such guidelines will facilitate the development of international educational programs that will support other ICU healthcare professionals. Overall, this proactive step prioritizes patients' needs ensuring a positive impact on global screening, assessment and management of PED.

## Author contributions

MD: Writing – original draft, Writing – review & editing. AG: Conceptualization, Supervision, Writing – review & editing.
